# Implementation of community-based screening program for risk factors of non-communicable diseases (NCDs) among the urbanized tribal population of West Bengal

**DOI:** 10.34172/hpp.025.44311

**Published:** 2025-11-04

**Authors:** Khushi Singhania, Sunom Merab Lepcha, Sembagamuthu Sembiah

**Affiliations:** ^1^Department of Community Medicine and Family Medicine, All India Institute of Medical Sciences, Kalyani, West Bengal, India; ^2^Department of Community Medicine, Hamdard Institute of Medical Sciences and Research, Hamdard Nagar, Delhi, India

**Keywords:** Indigenous populations, Mass screening, Non-communicable diseases, Preventive health services, Risk factors

## Abstract

**Background:**

Non-communicable diseases (NCDs) present a significant public health challenge, particularly among India’s tribal populations. This study aims to implement community-based screening to assess the current risk factors for NCDs in a tribal population, estimate the proportion at high risk using a Community-Based Assessment Checklist (CBAC), evaluate the program’s acceptability and fidelity, identify determinants of high-risk groups, and explore reasons for non-attendance at health centers by high-risk individuals.

**Methods:**

A mixed-methods cross-sectional study was conducted among 238 urbanized tribal individuals aged 30-60. Data were collected using a pre-designed, structured, and validated questionnaire in the local language and analyzed with SPSS version 26. Qualitative data were subjected to thematic analysis to provide a comprehensive understanding of the findings.

**Results:**

Of the participants, 88 (37%) were identified as high-risk, while 129 (54.2%) exhibited inadequate awareness of NCDs. The program demonstrated high acceptability (90.5%) but moderate fidelity (45.46%). Among the 88 high-risk individuals, 48 (54.54%) did not visit the primary health center (PHC). In-depth interviews with 10 randomly selected defaulters revealed key barriers, including financial and time constraints, inaccessibility of facilities, and a lack of seriousness and awareness regarding NCDs.

**Conclusion:**

The findings underscore an urgent need for multifaceted awareness-raising initiatives to improve NCD prevention and management in tribal populations. Enhanced education and accessibility to healthcare services are crucial for reducing the burden of NCDs in these communities.

## Introduction

 Non-communicable diseases (NCDs) represent a critical global public health challenge, accounting for 71% of all deaths worldwide and claiming 41 million lives annually. Notably, over 15 million individuals aged 30 to 69 succumb to NCDs each year, with 85% of these premature deaths occurring in low- and middle-income countries.^1^ Like many low- middle-income countries, India is also struggling to organize quality control care for a sizeable NCD-affected population, with 60% (5.8 million) of all deaths in the country attributed to NCDs, which mainly include coronary artery disease, stroke, hypertension, chronic respiratory diseases, cancers, and diabetes.^2^ NCDs account for more than half of the disability-adjusted life years (DALYs) in India.^3^

 The tribal population in India, as per the 2011 census, constitutes 8.6% of the nation’s total population and 5.8% of the population in West Bengal.^4,5^ Historically marginalized due to socio-economic and socio-demographic disadvantages, this group faces a high burden of NCD risk factors such as poor nutrition, tobacco and alcohol use, and hypertension.^4^ Recent studies indicate that 70% of NCD-afflicted individuals in various tribal districts of India die at home, with nearly one-fourth not receiving any treatment, underscoring the critical gaps in awareness and healthcare-seeking behavior.^6^ Frequently, the individuals from the tribal community tend to avoid accessible healthcare services, and despite various efforts, several barriers obstruct effective provision of healthcare services. Hence, it becomes imperative to recognize these gaps and take measures for their betterment.

 In response to the escalating NCD crisis, the Indian government launched the National Programme for Prevention and Control of Cancer, Diabetes, Cardiovascular Diseases and Stroke (NPCDCS). A key component of NPCDCS is the Community-Based Assessment Checklist (CBAC), designed to facilitate risk profiling and early diagnosis of NCDs by frontline workers. The CBAC aims to raise awareness about unhealthy practices, encourage healthy lifestyles, and improve access to timely healthcare interventions.^7^

 This study aimed to implement community-based screening to assess the prevalence of NCD risk factors among the urbanized tribal population of West Bengal. Additionally, it seeks to identify high-risk individuals, encourage their utilization of healthcare services provided at primary health centers (PHCs), and explore the barriers preventing them from seeking care. By addressing these objectives, the study aims to provide critical insights and recommendations for enhancing NCD prevention and management strategies in tribal communities. A mixed-methods approach was chosen to provide statistical insights and a contextual understanding of NCD risk factors and healthcare-seeking behavior among the urbanized tribal population. The quantitative phase identified high-risk individuals and established statistical associations, while the qualitative phase explored reasons for non-attendance at healthcare centers. This integration strengthens the study’s validity by capturing both prevalence and barriers affecting healthcare access.

## Materials and Methods

###  Study Design, Sampling and Tools

 This is a community-based cross-sectional study with a mixed-method design, conducted in an urbanized tribal population in a ward of Nadia district, West Bengal, with a total population of 2061 from September 2023 to January 2024. The study consists of two phases: The quantitative part consisted of screening for risk factors and assessing awareness of risk factors of NCDs using a questionnaire, and the qualitative part explored the reasons for not seeking care from health centers of high-risk referred cases through in-depth interviews.

####  Phase 1 Quantitative Phase

 The target group consisted of 878 individuals aged 30 to 60. Inclusion criteria were consent to participate, plans to remain in the area for the next 12 months, and proficiency in Bengali or Hindi. Exclusion criteria included individuals with diagnosed NCDs, pregnant women, and those who were mentally challenged or bedridden.

 A study on the urban village of Delhi with a similar methodology will be taken as a reference for calculating the sample size.^7,8^ The sample size for the study was calculated using the formula for proportions, i.e. N = (Z1−α/2)2 × p × (1 − p)/d2. Considering p as 0.17, 95% confidence interval, and absolute precision (d) of 5%, the required sample size was 217. With a 10% non-response rate, the final sample size was 238. In addition, as logistic regression analyses were planned, a priori power analysis was performed to assess adequacy for detecting associations. With the available sample size of 238, assuming an exposure and outcome prevalence of 37%, the study provided acceptable power to detect strong associations: approximately 73% power to detect an odds ratio (OR) of 2.0 and > 90% power for OR ≥ 2.5 (α = 0.05).

 Using the list of households provided by the ASHA worker as the sampling frame, each household with at least one resident aged 30–60 years was assigned a unique identifier, and simple random sampling (computer-generated random numbers) was used to select households until the required sample size of 238 was achieved. When more than one eligible individual was present in a household, one was chosen at random using the Kish grid. Household-level sampling was adopted as the ASHA records were organized by households rather than by individuals, and this approach was operationally efficient in community fieldwork. Selecting one individual per household reduced intra-household clustering, improved feasibility by requiring only a single visit per household, and maintained representativeness of the target population.

 Data was collected in Epi-Collect5 software (Centre for Genomic Pathogen Surveillance) through face-to-face interviews using a pre-designed, structured, and validated questionnaire, which consisted of three parts: 1) Socio-demographic and economic information (six questions like age, address, socio-economic status, etc.) 2) awareness regarding NCD and its risk factors. The NCD awareness questionnaire comprised 10 items with a 3-point Likert scale (Yes, No, Don’t Know).^9^ Those with a 50% or higher score were determined to have adequate awareness. 3) CBAC for risk assessment of NCDs.

 CBAC is an easy, non-invasive checklist and is used as a high-risk screening tool for NCDs. The checklist consists of three sections: The first section has questions on personal details. The second section deals with risk assessment and contains questions on lifestyle habits like smoking, alcohol consumption, physical activity, and family history of NCDs. Waist circumference measurements, which are categorized separately into male and female, are recorded in this section. A score of four and above is considered high risk for NCDs and should be referred to higher centres for further evaluation. The third section covers symptoms of NCDs. For this study, section two was used.

####  Phase 2 Qualitative Phase

**Figure 1:**
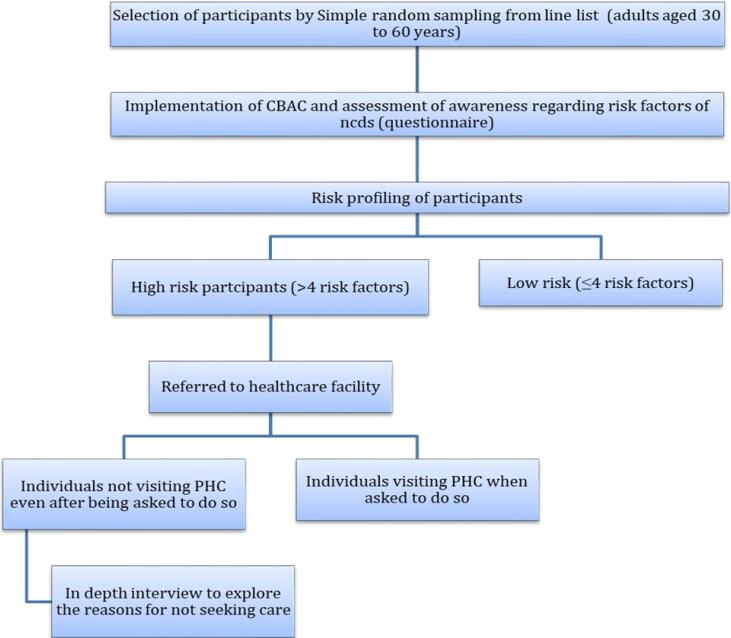
Flow diagram of participant selection, CBAC-based risk assessment, referral, and follow-up of study participants

 After the CBAC was implemented, participants who scored four or above on the CBAC were classified as high-risk and referred to a PHC for further evaluation and management (Figure 1). Cross-checks were performed with PHCs to assess follow-up compliance to determine the proportion of high-risk individuals who sought care. Among those who did not visit the PHC, ten were randomly selected for in-depth interviews to explore barriers to seeking care. The in-depth interviews were conducted using an interview guide developed by experts in community medicine.

###  Data Analysis

####  Quantitative Data

 Quantitative data were analyzed using IBM SPSS Statistics for Windows, Version 26.0 (IBM Corp., Armonk, NY, USA). Descriptive statistics for all variables were generated. The prevalence of risk factors for NCDs was expressed in proportions. For awareness on NCDs, those who had a score of 50% and above were considered to have adequate awareness. In the bivariate analysis, logistic regression was used to estimate the association between socio-demographic characteristics and levels of knowledge with risk category for NCDs at the 0.05 significance level. Assumptions for logistic regression, including independence of observations, absence of multicollinearity, linearity of continuous variables with the logit, and lack of influential outliers, were checked and found to be satisfied. In the bivariate analysis, crude ORs with 95% confidence intervals (CI) were calculated. Variables with *P* < 0.25 in the bivariate models were included in the multivariable logistic regression model to estimate adjusted ORs with 95% CI. Model fit was assessed using the Hosmer–Lemeshow goodness-of-fit test and Nagelkerke R^2^. There were no missing data for the study variables; therefore, all analyses were conducted on complete cases. Appropriate graphs and charts were prepared to represent the data.

####  Qualitative Data

 Qualitative data were analyzed using thematic analysis. Interviews were recorded, transcribed verbatim, and coded independently by two researchers. Thematic analysis followed Braun and Clarke’s six-step framework, which includes familiarization with the data, generating initial codes, developing themes, reviewing themes, defining and naming themes, and finally writing up the findings. This process involved deep immersion in the data to extract meaningful interpretations. Themes and codes were generated through a systematic and detailed reading of the transcripts. NVivo software was used to facilitate coding and theme identification. To ensure reliability and validity, inter-rater reliability was maintained through double coding and discussion resolution.

## Results

 This study was conducted among 238 individuals with a mean age of 41.6 ± 8.13 years, ranging from 30 to 60 years. Two-thirds (67%) of the participants were females, 109 (45.8%) of the participants belonged to the age group of 30-39 years, 197(80.3) were educated, 118(49.8) were unskilled, 138(58%) belonged to the lower socio-economic group, 154(64.7%) belonged to nuclear family (Table 1).

**Table 1:** Distribution of study participants according to socio-demographic profile (N = 238)

**Socio-demographic profile**	**No. (%)**
Age	
< 40 years	109 (45.8)
≥ 40 years	129 (54.2)
Sex	
Male	88 (37.0)
Female	150 (63.0)
Education	
Illiterate	63 (26.5)
Educated	175 (73.5)
Occupation	
Unskilled	185 (77.4)
Others	53 (22.6)
Socio-economic status (as per BG prasad scale 2024)	
Upper middle/middle	100 (42.0)
Lower	138 (58.0)
Type of family	
Nuclear	84 (35.3)
Joint	154 (64.7)

###  Prevalence of NCD Risk Factors

 Out of 238 participants, 150 (63%) had a total score equal to or less than 4, indicating they were at low risk. On the other hand, 88 (37%) of the individuals had a score of more than 4, thus suggesting they were at high risk.All the participants had at least 1 risk factor, but none reached the maximum possible score of 12. The total scores ranged from a minimum of 1 to a maximum of 8, with a mean score of 3.96 ± 1.83 (Table 2).

**Table 2:** Prevalence of risk factors for NCDs as per CBAC (N = 238)

**Risk factors**	**No. (%)**
Age (y)	
30-39 years	109 (45.8)
40-49	79 (33.2)
50-60	50 (21.0)
History of tobacco consumption	
Never	160 (67.2)
Used to consume in the past	20 (8.4)
Daily	58 (24.4)
History of alcohol consumption	
No	214 (89.9)
Yes	24 (10.1)
Waist circumference (cm)	
< 81 in females and < 91 cm in males	94 (39.5)
81-90 in females and 91-100 in males	117 (49.2)
≥ 91 in females and ≥ 101 in males	27 (11.3)
Physical activity	
At least 150 minutes in a week	158 (66.4)
Less than 150 mins in a week	80 (33.6)
Family history of NCDs	
Yes	58 (24.4)
No	180 (75.6)

###  Awareness Regarding Risk Factors for NCDs

 Among 238 participants, almost half 129(54.2%) had poor awareness (scored < 5) with a mean score of 4.14 ± 3.73. Seventy (30.7%) participants scored the minimum score of 0, whereas only 22(9.2%) could achieve the maximum score of 10. Around 60% informed that the screening helps in diagnosing NCDs. Females were more aware of NCD than males.

###  Acceptability and Fidelity of Screening

 Around 263 individuals were approached, out of whom 238 agreed to participate. Thus, the sample size was achieved, indicating an acceptability rate of 90.5%. 88(37%) participants were in the “high-risk group”, out of whom, 40 failed to visit the PHC, showing a fidelity rate of 45.46%.

###  Factors Associated with High Risk for NCD

 Among sociodemographic characteristics, males and participants from nuclear families were significantly more likely to be classified as high-risk for NCDs. Other sociodemographic factors, including education, occupation, and socio-economic status, were not significantly associated with high-risk status. The logistic regression model including sociodemographic variables explained 18% of the variance in high-risk status (Nagelkerke R^2^ = 0.18), and the Hosmer–Lemeshow test indicated a good fit (*P* = 0.60).

 Regarding behavioral and medical risk factors, participants aged ≥ 40 years, those who used tobacco, consumed alcohol, had high waist circumference, or had a family history of NCDs were significantly more likely to be classified as high-risk. The multivariable logistic regression model, including these risk factors, explained 52% of the variance in high-risk status (Nagelkerke R^2^ = 0.52) and demonstrated good model fit (Hosmer–Lemeshow test, *P* = 0.746).

 These findings highlight the critical role of age, lifestyle behaviors (tobacco and alcohol use), waist circumference, and family medical history in identifying individuals at high risk for NCDs (Tables 3 and 4).

**Table 3:** Association of sociodemographic profile with “high risk” group for NCDs (N = 238)

**Sociodemographic Profile**	**Low Risk (n, %)**	**High Risk (n, %)**	**Crude OR (95% CI)**	***P*** ** value**	**Adjusted OR (95% CI)**	***P*** ** value**
Sex						
Male	45 (51.1)	43 (48.9)	2.23 (1.29–3.84)	0.004*	3.43 (1.72-7.03)	0.001*
Female	105 (70.0)	45 (30.0)	1	–	1	–
Education						
Educated	111 (63.4)	64 (36.6)	1	–		
Illiterate	39 (61.9)	24 (38.1)	1.06 (0.58–1.93)	0.829		
Occupation						
Unskilled	117 (63.2)	68 (36.8)	1	–		
Skilled	33 (62.3)	20 (37.7)	1.04 (0.55–1.95)	0.903		
Socio-economic Status						
Lower	87 (63)	51 (37)	1	–		
Upper/Middle	63 (63)	37 (37)	1.00 (0.59–1.70)	0.995		
Type of Family						
Joint	105 (68.2)	49 (31.8)	1	–	1	–
Nuclear	45 (53.6)	39 (46.4)	1.85 (1.07–3.20)	0.026*	1.96 (1.11-3.46)	0.020*

OR, odds ratio; CI, confidence interval. * Statistically significant (*P* < 0.05).

**Table 4:** Association of risk factors and awareness regarding NCD with “high risk” group for NCDs (N = 238)

**Variable**	**Low Risk (n, %)**	**High Risk (n, %)**	**Crude OR (95% CI)**	***P*** ** value**	**Adjusted OR (95% CI)**	***P*** ** value**
Age						
< 40	96 (88.1)	13 (11.9)	1	-	1	-
≥ 40	54 (41.9)	75 (58.1)	10.26 (5.36–20.93)	< 0.001*	12.3 (6.2–24.5)	< 0.001*
Tobacco consumption						
Never/past users	136 (75.6)	44 (24.4)	1	-	1	-
Daily users	14 (24.1)	44 (75.9)	9.7 (4.8-19.8)	< 0.001*	6.8 (3.5–23.2)	< 0.001*
Alcohol consumption						
No/Occasional	146 (68.2)	68 (31.8)	1	-	1	-
Yes	4 (16.7)	20 (83.3)	10.74 (3.89–37.99)	< 0.001*	4.5 (1.7-42.1)	0.002*
Waist circumference						
< 81 cm (females) / < 91 cm (males)	71 (75.5)	23 (24.5)	1	-	1	-
≥ 81 cm / ≥ 91 cm	79 (54.9)	65 (45.1)	2.54 (1.45–4.57)	0.001*	3.2 (1.7–6.0)	< 0.001*
Physical activity						
≥ 150 min/wk	109 (69.0)	49 (31.0)	1	-		
< 150 min/wk	41 (51.2)	39 (48.8)	2.12 (1.22–3.69)	0.008*	2.8 (1.5–5.2)	0.001*
Family history of NCDs						
No	135 (75.0)	45 (25.0)	1	-		
Yes	15 (25.9)	43 (74.1)	8.60 (4.46–17.40)	< 0.001*	5.5 (3.0–10.1)	< 0.001*
Awareness regarding NCD						
Adequate	69 (63.3)	40 (36.7)	1	-		
Inadequate	81 (62.8)	48 (37.2)	1.02(0.6-1.7)	0.935		

OR, odds ratio; CI, confidence interval. * Statistically significant (*P* < 0.05).

###  Results of In-Depth Interviews with the Defaulters

 Eighty-eight (37%) of the study participants were labeled as “high-risk” and were referred to the PHCs, and a referral slip was given to them. Out of 88 participants, around 48 (54.54%) failed to visit the PHC (defaulters). Ten defaulters were randomly selected, and in-depth interviews (IDIs) were conducted to explore the reasons for not seeking care from health centers. In this study, three main themes emerged, mainly time and financial constraints, lack of awareness, and inaccessibility.

 The participants, primarily females, stated that they have no time for going to PHCs as their children are too young to be left alone at home, and nobody can take care of them. Some participants, who were daily wage earners and office workers, worked for the entire day and had no time to visit hospitals.

 “*With work commitments and personal responsibilities, finding time to make an appointment and travel to the center is extremely challenging.”*

 “*I can’t bring my children to the appointments. The well-being and safety of my children are my top priority, and without proper childcare, I am unable to make hospital visits.”*

 The study participants mainly belonged to lower and middle socio-economic backgrounds. For many of them, the tests prescribed by doctors, even at government hospitals, are too expensive when they are struggling to meet their daily basic requirements. They suggested that the basic tests and medications should be free for those who cannot afford them.

 “*The costs associated with diagnostic tests and necessary medications are beyond my financial means. Despite understanding the importance of regular health check-ups, the high expenses make it impossible for me to afford the care I need.”*

 Most of the respondents were unaware that NCDs, such as diabetes and hypertension, are asymptomatic in the early stages and can have severe complications in later stages. Early diagnosis and treatment can prevent these complications. This was mainly due to a lack of education and awareness. They suggested that steps should be taken and more awareness-related programs should be started by the government concerning NCDs, which will be helpful for people like them.

 “*I didn’t visit the healthcare center because I had no symptoms and therefore considered my condition to be not serious. I assumed that if something were wrong, I would feel it or see some signs.”*

 Some participants did not know how to use it properly for registration at healthcare centers. Individuals also complained of long waiting hours at PHCs and denial of treatment even after waiting, as the center closed by the time their turn came. This indicates poor management and infrastructure, which should be taken care of. They suggested that setting up more centers or providing services on a camp basis may be useful for them.

 “*I don’t know how to register for the services. The process seems confusing and I haven’t found clear information or guidance on how to get started.”*

 “*Every time I go, I end up waiting in line for hours, and by the time my turn comes, the center has already closed.”*

## Discussion

 This community-based study among an urbanized tribal population in West Bengal provides important insights into the burden of NCD risk factors, awareness levels, and barriers to healthcare access. More than one-third of the participants were at high risk for NCDs based on the CBAC tool, while more than half had inadequate awareness of NCD risk factors. Although the screening program demonstrated high acceptability (90.5%), fidelity was considerably lower (45.4%), highlighting a disconnect between risk identification and subsequent healthcare utilization. A study in Haryana focused on NCD screening and highlighted significant acceptability but lower fidelity rates, echoing the issues identified in our research.^10^

 Our findings are consistent with previous studies in India that documented a rising burden of NCD risk factors among tribal and marginalized communities.^11,12^ In Delhi, Khokhar et al^7^ reported that 17.2% of individuals were at high risk based on CBAC, though the prevalence was lower than in our study, possibly due to differences in population characteristics and scoring criteria. A STEPS survey from Haryana observed a similarly high prevalence of behavioral risk factors, particularly tobacco and alcohol use, which were also significant predictors in our study.^13^ Studies from Assam and Darjeeling among tribal populations have shown even higher tobacco and alcohol consumption, suggesting that local cultural practices and accessibility influence behavior.^14,15^

 Increasing age and a positive family history of NCDs are among the most important non-modifiable risk factors. In our study, 58.1% of individuals over 40 years and 74.1% of individuals with a positive family history were at higher risk, while the overall presence of family history was 24.4%, similar to a study conducted in Delhi but much lower than that in Haryana, where more than 70% of participants had a positive family history.^7,13^ This difference may be due to the inclusion of individuals already diagnosed with NCDs in the Haryana study, as genetic makeup and similar environmental and behavioral characteristics play an important role in the development of NCDs.

 Physical inactivity and abdominal obesity are important modifiable risk factors contributing to NCDs. In the present study, 60.5% of participants had abdominal obesity, and 33.6% were physically inactive, similar to Khokhar et al.^7^ However, studies conducted by Bhar et al,^15^ Misra et al,^14^ and Tushi et al^16^ reported lower prevalence as both urban and rural populations were surveyed, with people more involved in outdoor and cultivation-related activities. Surveys in Kerala and Haryana found females to be more physically active than in our study, possibly due to socio-cultural differences, higher literacy rates, better opportunities, and greater health awareness.^13,17,18^ In contrast, an urban Puducherry population study reported much higher coronary risk factor prevalence, highlighting stark urban–tribal differences. These comparisons emphasize the importance of context-specific health promotion strategies that account for lifestyle transitions in urbanized tribal communities.^19^

 Among behavioral risk factors, current tobacco and alcohol consumption was 24% and 10.1%, respectively, similar to studies in Delhi and Haryana.^7,13^ Higher consumption has been observed in Nepal and Siliguri, likely due to cultural practices, local farming, and cross-border tobacco trafficking.^15,20^ In contrast, Kerala reported lower tobacco use (7.9%), potentially reflecting higher literacy and awareness, while lower alcohol consumption in Nepal and Nagaland may be influenced by cultural attitudes discouraging alcohol use.^16,17,20^

 Awareness of NCDs was poor in our population, with more than half unable to correctly identify risk factors, aligning with findings from Rwanda and Tanzania, where low levels of NCD knowledge were reported, particularly among populations without prior exposure to health systems.^9,21^ In Kerala, higher literacy and health literacy were associated with greater awareness, highlighting the role of education and IEC campaigns in improving community engagement.^17^

 The IDIs provided insight into reasons for low healthcare utilization despite high risk. Key barriers included lack of time, awareness, funds, and inadequate facilities for an increasing population. Similar findings were reported by Sangar et al^22^ and Elias et al,^2^ where participants did not seek treatment due to being uninformed or unconcerned about adverse outcomes. Unlike these studies, distance or lack of nearby medical facilities was not reported in our study, as PHCs were accessible. Addressing these gaps requires making facilities more affordable and increasing the number of government-provided services.^23-25^

 These findings underscore the need for multi-pronged interventions. First, culturally tailored awareness campaigns should be delivered through trusted community channels such as ASHAs, schools, and religious gatherings, as evidence shows that community engagement and peer education can significantly improve NCD knowledge and behavior change.^11,26,27^ Second, health system strengthening is required to ensure functional, affordable, and acceptable referral pathways, as demonstrated by Ayushman Bharat Health and Wellness Centres in India and WHO’s Package of Essential NCD Interventions (PEN) in LMICs.^4,28^ Third, digital health innovations, including telemedicine and electronic health cards, can bridge gaps in fidelity by reducing logistical barriers and enabling follow-up.^29,30^

 Integrating CBAC-based risk screening with routine health promotion activities may improve early detection of NCDs in tribal communities. However, screening alone is insufficient; bridging the “know–do” gap requires addressing both individual-level barriers (awareness, motivation) and system-level barriers (accessibility, affordability). Regular health camps, mobile clinics, and targeted subsidies for diagnostic tests may enhance service utilization and promote better health outcomes

## Conclusion

 In this study, 88 (37%) participants had a high risk for NCDs, and 129 (54.2%) had inadequate awareness regarding NCDs. The acceptability and fidelity rate was 90.5% and 45.46%, respectively. The proportion of inadequate awareness among males and females was 50% and 56.6%, respectively. Participants from the nuclear families, those aged 40 years and above, tobacco and alcohol users, and those with a positive family history of NCDs showed a significantly higher risk of being in the high-risk group On conducting IDIs to explore the reasons for not visiting healthcare facilities, reasons like time constraints, financial issues, lack of awareness, and poor accessibility were stated.

 Awareness activities regarding NCDs should be frequently conducted. IEC (Information Education and Communication) campaigns may be conducted in different settings, such as schools, workplaces, religious gatherings, etc. Healthcare facilities should be made more affordable and accessible and researchers/policy makers should try to make an effort to find the reasons for low fidelity. Electronic Health cards can serve as a centralized repository that can be seamlessly accessed and utilized by healthcare providers across different states thus offering a unified platform for healthcare management across the different states in India. Through this, the healthcare providers can identify individuals at a higher risk and recommend appropriate management.

## Competing Interests

 The authors declare that they have no competing interests.

## Data Availability Statement

 The datasets generated during the current study contain confidential information on participants and are not publicly available due to ethical restrictions. However, de-identified data may be made available from the corresponding author on reasonable request.

## Ethical Approval

 Ethical approval was obtained from the Institutional Ethics Committee (IEC) vide Letter No. IEC/AIIMS/Kalyani/Meeting/2023/079-R. Participation was entirely voluntary and anonymous. All participants were fully informed about the nature and aims of the study, and before any subject was included in the study, their informed written consent was obtained.
